# Rapid Displacement of Dengue Virus Type 1 by Type 4, Pacific Region, 2007–2009

**DOI:** 10.3201/eid1601.091275

**Published:** 2010-01

**Authors:** Dong-sheng Li, Wen Liu, Aurélie Guigon, Candice Mostyn, Richard Grant, John Aaskov

**Affiliations:** Queensland University of Technology, Brisbane, Queensland, Australia (D. Li, J. Aaskov); Australian Army Malaria Institute, Brisbane (W. Liu, C. Mostyn, R. Grant, J. Aaskov); Institut Pasteur, Noumea, New Caledonia (A. Guigon)

**Keywords:** Dengue, emerging disease, Pacific region, viruses, dispatch

## Abstract

Since 2000–2001, dengue virus type 1 has circulated in the Pacific region. However, in 2007, type 4 reemerged and has almost completely displaced the strains of type 1. If only 1 serotype circulates at any time and is replaced approximately every 5 years, DENV-3 may reappear in 2012.

During the past 10–15 years in the Pacific island nation states, sustained transmission of only 1 serotype of dengue virus (DENV) has occurred at any given time (*1*)*.* This single serotype is in marked contrast to all 4 serotypes that cocirculate in many countries in Southeast Asia where dengue is endemic. During 1997–2000 in the Pacific region, the serotype recovered from patients was almost exclusively DENV-2, but during 2000–2001, <1 year, DENV-2 was displaced by multiple genotypes of DENV-1 (*2*). We describe rapid replacement of DENV-1 by DENV-4 during 2008.

## The Study

**Figure 1:**
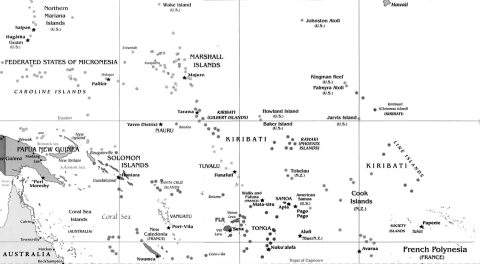
Island nation states of the Pacific region.

In May 2008, an outbreak of a dengue-like illness began on the island of Tarawa in Kiribati (Figure 1). Immunochromatographic and ELISA assays (PanBio, Brisbane, Queensland, Australia) detected anti–dengue virus immunoglobulin (Ig) M or high titers (>1,280) of anti–dengue virus IgG in serum from 5 of 18 patients. DENV-4 transmission had not been reported in the Pacific region for >1 decade; however, after serum was cultured with *Aedes albopictus* C6/36 cells, DENV-4 was recovered from 5 of the 13 serum samples from patients who had no detectable anti-DENV IgG or IgM (*2*)*.*

In July 2008, a similar outbreak began in Samoa. Serum from 87 of 469 patients with suspected dengue contained anti-DENV IgM or high titers of anti-DENV IgG (ELISA; PanBio). Serum from 7 of the 87 patients with anti-DENV IgM contained no detectable anti-DENV IgG, suggesting a primary infection. DENV-4 was recovered from 42 of the 382 seronegative patients when serum was cultured with C6/36 cells.

From June 2007 through February 2008 in Tonga, small numbers of dengue cases, confirmed by ELISA, had been reported; the only DENV isolates recovered from these patients were 4 isolates of DENV-1 (J. Aaskov, unpub. data). However, in December 2008 and January 2009, only 4 DENV-4 isolates were recovered from 55 serum samples collected from patients with suspected dengue in Tonga.

In November 2008 in New Caledonia, DENV-4 transmission was detected after the virus had been introduced by residents returning from Vanuatu; and in February 2009, DENV-4 transmission was detected in French Polynesia after it had been introduced there by travelers from New Caledonia. In June 2009, DENV-1 and DENV-4 were cocirculating in New Caledonia and French Polynesia.

DENV-4 was reportedly recovered from a traveler returning to Taiwan from the Solomon Islands in 2007 (*3*) and from a resident of the Solomon Islands in April 2008 (Alyssa Pyke, pers. comm.). DENV-1 had been circulating in the Solomon Islands until at least 2002, preceding the 2003 arrival of a multinational peacekeeping force composed of persons from Australia, Papua New Guinea, New Zealand, Fiji, and Tonga. The chronology of these and other reports of dengue outbreaks involving DENV-4 are shown in the Table.

**Table:** Chronology of appearance of dengue virus type 4, Pacific region

Date of appearance	Country	Source/reference
2007	Solomon Islands	(*3*)
2008 Apr	Nauru	T. Bryar, pers. comm.*
2008 May	Kiribati	This study
2008 Jul	Samoa	This study
2008 Jul	American Samoa	T. Bryar, pers. comm.*
2008 Jul	Palau	T. Bryar, pers. comm.*
2008 Aug	Cook Islands	T. Bryar, pers. comm.*
2008 Aug	Fiji	T. Bryar, pers. comm.*
2008 Aug	Niue	T. Bryar, pers. comm.*
2008 Oct	Vanuatu	T. Bryar, pers. comm.*
2008 Dec	Tonga	This study
2008 Nov	New Caledonia	This study
2009 Feb	French Polynesia	T. Bryar, pers. comm.*
2009 Jun	Chile (Easter Island)	ProMED-mail†

*Western Pacific Regional Office, World Health Organization. †www.promedmail.org/pls/otn/f?p=2400:1202:1356950393966944::NO::F2400_P1202_CHECK_DISPLAY,F2400_P1202_PUB_MAIL_ID:X,78506

**Figure 2:**
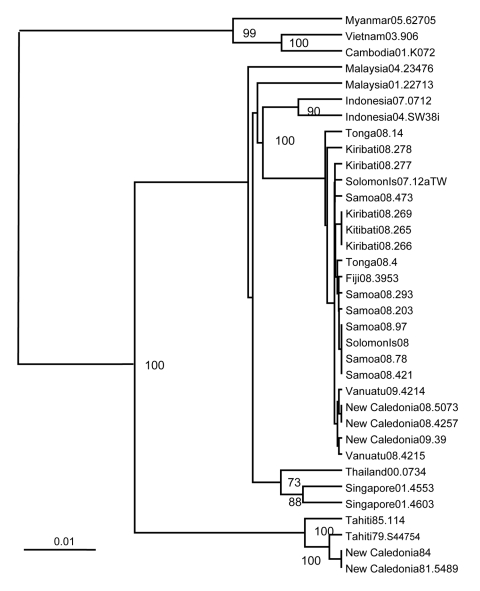
Phylogenetic relationships between the envelope (E) protein genes of dengue type 4 viruses recovered from patients from Pacific island nation states during 2007–2009 outbreaks and from dengue type 4 viruses from Southeast Asia and earlier outbreaks in the Pacific region. Bootstrap values are shown for key nodes. Scale bar represents 0.01 nt changes per site.

During some of these outbreaks, the envelope (E) protein genes of DENV-4 recovered from patients were amplified by reverse transcription–PCR (*2*) by using forward primer 5′-GGATTCGCTCTCTTGGCAGGATTTATG-3′ and reverse primer 5′- GCTTCCACACTTCAATTCTTTCCCACTCCA-3′, corresponding to regions in the premembrane and nonstructural protein gene 1, respectively, and the consensus nucleotide sequences of the resultant cDNA determined by Dye Terminator Cycle Sequencing on an automated sequencer (ABI Prism, Australian Genome Research Facility, Brisbane, Queensland, Australia). Sequencing was performed by using the primers above as well as 5′-AACACAGCATGGGACAACAGT-3′ and 5′-GACTCAAACATCTTACCAATGGAG-3′. Sequences were analyzed by using ClustalW, Seqboot, DNADist, Kitsch, and Consense software (www.angis.org.au) from the Australian National Genome Information Service of the University of Sydney. Phylogenetic analyses (Human Research Ethics Approval QUT-0700000910) of the nucleotide sequences of the E genes of DENV-4 that we recovered from patients in Kiribati, Samoa, and Tonga and those of strains of DENV-4 recovered by others showed that all isolates from this recent outbreak in the Pacific were closely related but distinct from other DENV-4 isolates for which sequences were available (Figure 2), including isolates recovered in the Pacific region during the 1970s and 1980s.

The chronology of the recovery of DENV-4 from patients in the region and the phylogenetic analyses suggest that DENV-4 was introduced from Indonesia/Malaysia into the Pacific region, possibly into the Solomon Islands, sometime before 2007. The 3 genotypes of DENV-1 responsible for the earlier outbreak also originated in Southeast Asia (Philippines, Malaysia, Myanmar/Thailand) (*2*)*.* The relative genetic homogeneity of the DENV-4 recovered during this most recent outbreak in the Pacific region (24 variable nucleotide sites in the E genes of 20 isolates resulting in 9 variable amino acid sites) suggests introduction of a single genotype rather than introduction of multiple genotypes and to different locations, as was the case with DENV-1 (*2*). The E proteins of all recent DENV-4 isolates from the Pacific region had isoleucine at position E365 rather than the threonine that was found at this position in earlier DENV-4 isolates. All Pacific region isolates except DENV4 Kiribati08.278 also had isoleucine at E335 rather than valine, which was found at this position in most earlier isolates. These 2 aa changes occurred in a region of domain III of the E protein of flaviviruses rich in epitopes recognized by serum from dengue patients and by neutralizing monoclonal antibodies (*4**–**6*); they occurred adjacent to the change at E390 in DENV-2, which was associated with the appearance of dengue hemorrhagic fever in South America (*7*).

## Conclusions

Outbreaks of dengue in the Pacific region are initiated by the introduction of DENV, usually from Southeast Asia, but the populations of most Pacific island nation states are too small to sustain transmission of a single DENV serotype for >4–5 years. The interisland mobility of the human population in this region ensures rapid spread of any newly introduced viruses.

That the spread of dengue virus serotypes through the Pacific should be so synchronized is remarkable. This synchronization may reflect the relatively small populations of most island states (≈250,000 residents), high attack rates, and a high birth rate (≈30% of the population is <14 years of age). If only 1 DENV serotype circulates at any time, and serotype replacement occurs approximately every 5 years, these data suggest that ≈30% (75,000) of 250,000 susceptible hosts are sufficient in these settings to support a serotype replacement and that DENV-3 may reappear in the Pacific island states in ≈2012.

At this stage of study, data are insufficient for drawing conclusions about a role for the amino acid changes at E335 and E365 in the reemergence of DENV-4 in the Pacific region. There may be value in delineating the factors that appear to enable multiple DENV serotypes to circulate in urban areas of more developed Pacific nations (e.g., French Polynesia, New Caledonia, Australia) but that appear to prevent cocirculation of DENV serotypes in nations that are less developed but rapidly becoming urbanized. Such a study, however, would require more robust and comprehensive dengue surveillance programs than exist in many of these nations.
