# Correction: Vol. 16, No. 1

**DOI:** 10.3201/eid2305.C12305

**Published:** 2017-05

**Keywords:** errata, erratum, correction

The name of author Emerald Stewart was omitted from Rapid Displacement of Dengue Virus Type 1 by Type 4, Pacific Region, 2007–2009 (D. Li et al.). The article has been corrected online (https://wwwnc.cdc.gov/eid/article/16/1/09-1275_article).

